# 

**Published:** 1898-08

**Authors:** 


### CONTENTS.

| | PAGE |
|---|---|
| **ORIGINAL ARTICLES:** | |
| Immunity. By A. T. PETERS, D.V.M. | 515 |
| The Internal Chicken-mite (Cytodites Nudus Vizioli). By E. V. WILCOX | 523 |
| Veterinary Preparatory Education. By ROBERT ROBB, V.S., M.D. | 526 |
| A Method of Generating and Administering Medicated Steam to Horses. By R. C. MOORE, D.V.S. | 528 |
| The Value and Use of the Thermo-Cautery. By GEORGE P. TUCKER, V.S. | 529 |
| **ABSTRACTS FROM FOREIGN JOURNALS:** | |
| Cheap Practitioners | 531 |
| The Question of the Extermination of Cattle-tuberculosis | 532 |
| Physiological and Clinical Investigation of a White Dog with Blue Eyes | 533 |
| Proposed Law for the Extermination of Cattle-tuberculosis in Denmark | 534 |
| Statistics Regarding Tuberculosis in the Prussian Slaughter-houses | 534 |
| **SELECTIONS:** | |
| The Microbe of Pleuro-pneumonia. By MESSIEURS NOCARD and ROUX | 535 |
| The Scope of Pasteur's Work | 542 |
| How One of our Editors is Regarded by the Lay Press | 543 |
| **THERAPEUTIC NOTES** | 546 |
| **REPORTS OF CASES:** | |
| Operation for Removal of Post-Nasal Fibroid Tumor. By C. J. SIHLER, V.S. | 550 |
| Meso-neurectomy | 552 |
| Peroneus Tenotomy | 553 |
| Posterior Tibial Neurectomy | 553 |
| Cunean Tenectomy | 553 |
| Arytenoideraphy | 553 |
| **AMONG MISSOURI VALLEY VETERINARIANS** | 553 |
| **EDITORIALS:** | |
| At Omaha | 557 |
| Aid the Red-Cross Organizations | 557 |
| The Colleges of the Central West | 558 |
| The Programme is on Trial | 558 |
| Tuberculin Test in New York State | 559 |
| Treatment of Milk-fever in New Jersey | 559 |
| The Army Bill | 559 |
| **ARMY DRIFT** | 560 |
| **U. S. V. M. A. MEETING, 1898, OMAHA, SEPTEMBER 6, 7, 8** | 564 |
| **SOCIETY PROCEEDINGS** | 566 |
| **AMONG THE COLLEGES** | 575 |
| **PERSONALS—POINTS** | 578–582 |

